# Treatment of Spinal Tuberculosis of GATA Type III: Primary Posterior Debridement Combined with Osteotomy Parallel to the Endplates for Reconstruction

**DOI:** 10.1111/os.12650

**Published:** 2020-04-23

**Authors:** Yi Feng, Yu‐shan Wang, Jia Lv, Zhi Lv, Bin Zhao, Sheng Zhao, Cai‐tong Cheng

**Affiliations:** ^1^ Department of Orthopaedics The Second Hospital of Shanxi Medical University Taiyuan China

**Keywords:** Spinal tuberculosis, Kyphosis, Osteotomy, Orthopaedic procedure

## Abstract

To evaluate the curative effect of one‐stage posterior debridement and osteotomy parallel to the endplates for reconstruction, deformity correction, and tuberculosis control on treating the spinal tuberculosis of graded GATA III. From July of 2012 to December of 2017, there were 36 cases from the Second Hospital of Shanxi Medical University with thoracic and lumbar tuberculosis graded GATA III, in which we used osteotomy parallel to the endplates and reconstruction for treatment,16 for males and 20 for females. The local Cobb angles of kyphosis of all patients are greater than or equal to 20.The age varied from 28 months to 72 years with an average of 38.8 years. There were 15 cases of thoracic segment, 12 cases of thoracolumbar segment (T_11_‐L_2_), 9 cases of lumbar segment. Preoperative results of ASIA were 3 cases of grade C,5 cases of grade D and 28 cases of grade E with an average kyphosis Cobb angle of 37.21 ± 3.28. The visual analogue scale(VAS) scores preoperatively were 0‐8 points (averaged 5.58 ± 1.66 points). All the patients had paraspinal abscesses. After completing the preoperative examinations and evaluations, the osteotomy parallel to the endplates and reconstruction were executed. We made a statistical analysis of the Cobb angles, visual analogue scale(VAS) scores, erythrocyte sedimentation rate (ESR), C‐reaction protein(CRP), and ASIA grades before and after the surgery. The following‐up time varied from 12 to 24 months, with an average of 18 months. The VAS score improved from 5.58 ± 1.66 before the surgery to 3.25 ± 0.92 one month after the surgery and 2.12 ± 0.73 at the last follow‐up. The Cobb angles decreased from 37.21° ± 3.28° before the surgery to 5.72°± 2.66° one month later and 5.99° ± 1.92° at the last follow‐up. The ESR decreased from 55.34 ± 1.72 mm/1 h before the surgery to 28.22 ± 3.76 mm/1 h one month later and 11.54 ± 0.46 mm/1 h at the last follow‐up. The CRP decreased from 35.22 ± 2.46 mg/L before to 12.67 ± 2.82 mg/L and 4.50 ± 2.11 mg/L at the last follow‐up. The results of the last ASIA grades were 1 case of grade D and 35 cases of grade E. The one‐stage posterior debridement and osteotomy parallel to the endplates for patients with spinal tuberculosis of graded GATA III are not only beneficial to spinal reconstruction, but also obtain ideal reconstuction effects.

## Introduction

Spinal tuberculosis (ST) is the most common bone tuberculosis. Its incidence has increased in recent years especially in developing countries^1^. Approximately 3%–5% of cases had musculoskeletal involvement in tuberculosis; nearly 50% of these patients had spinal involvement. Spinal tuberculosis is common in children and teenagers, but the morbidity in old people is higher and higher nowadays. It is still the important cause of high disability and fatality rates in China. It is widely accepted that spinal tuberculosis is derived from the pulmonary and genitourinary lesions through blood transmission and direct diffusion. However, it is worth noting that spinal tuberculosis often coexists with some nonspecific infection, which makes performance and drug therapy different. Patients often have an indolent disease course with back pain as the major symptom. The ESR (Erythrocyte Sedimentation Rate) and CRP (C‐Reactive Protein) values are commonly high with the temperature and white blood cell count in the normal range. These characters make the diagnosis difficult and then the imaging data becomes a very important clue. Lumbar vertebra is the most common site that mycobacterium tuberculosis invades because of the high vascularization and frequent activity. And thoracic vertebra is the second most common site. Spinal tuberculosis is classified into three types on the basis of the damaged part of the vertebrae: (i) Borderline type: the damaged area is mainly focus on the upper and lower margin with intervertebral disc and adjacent vertebra involvement. This type is common in adults and generally located in lumber vertebra; (ii) Center type: The damaged area is mainly focused on the central part of the vertebral body which is often compressed into wedges. This type is common in children and generally located in thoracic vertebra; (iii) Accessory type: The damaged area is mainly focused on pedicle, vertebral plate and spinous process. This type is very rare.

The vertebral tuberculosis lesions often involve the anterior and middle column, which is easy to cause kyphosis and spinal cord compression. As a result, the incidence of neurological dysfunction is up to 10%–41%^2^. For severe cases, surgery is necessary, which can significantly shorten the course of disease, reduce the usage of anti‐tuberculosis drugs, and prevent paralysis. Most of ST is in thoracolumbar segments, that can lead to deformity such as kyphosis. Bone grafted without instrumentation was often unsatisfactory in correcting or preventing the progression. However, there has been a significant evolution in the treatment of spinal tuberculosis during the past few decades. Posterior pedicle screw system has been applied more often as a revolutionary technique for correcting deformity and stabilizing. The anterior approach debridement and intervertebral fusion has been recognized as the “golden standard” treatment for spinal tuberculosis, while anterior approach has defects such as thoracic or abdominal complication, unsatisfactory deformity correction, and great trauma^3^. The combined anterior and posterior approach can obtain ideal deformity correction, but longer operation time, much more blood loss, and trauma make it an abandoned method. The primary surgery of posterior approach has been increasingly recognized and applied more often in clinical practice due to its small trauma, fewer complications, and ideal result^4^, ^5^. Based on clinical performance and imageological examination, GATA classification system proposes four types of spina tuberculosis (Type IA, Type IB, Type II, and Type III) and seven evaluative criterion (abscess formation, degeneration and destruction of the vertebral disc, destruction and collapse of the vertebral body, spinal stability, kyphosis, sagittal plane index, deformity of the neurological system). Spinal tuberculosis of GATA III is the severest type that destructs vertebra or intervertebral space which leads to a kyphosis more than 20°. At the same time, it destroys the spinal stability and produces mass paravertebral absesses with or without neurologic deficit. This kind of lesion is the heavest type that always needs surgery. Except for the compression made by absesses, granulation tissue, and caseous necrosis, the disability by subluxation as well as the kyphosis by spinal broken can make spinal cord and nerve roots injured. So the procedure of decompression, stability reconstruction, and deformity correction based on chemotherapy have to be necessary.

Since spinal tuberculosis of GATA III has kyphosis deformity, the posterior approach surgery has obvious advantages in deformity correction. However, in terms of reconstruction, it is more difficult to implant titanium mesh into the focus. What's more, since the focus is mostly irregular and located in front of the intervertebral space, the height, contact surface, and stability of titanium mesh are always difficult to meet the ideal requirements. Although non‐structural bone grafting and profile‐shaped titanium mesh have been used^6^, ^7^, small scope of lesion clearance, residual of sub‐healthy tissues, and poor height supported are its inevitable flaws. For some cases with serious kyphosis and long course of disease, the orthopaedic reconstruction is even harder. Thus, we adopted primary posterior debridement combined with osteotomy parallel to endplates for deformity correction to treat spinal tuberculosis of GATA III and achieved satisfactory curative effect.

The purpose of this study was to propose a creative surgical method which breaks the limitation of the traditional posterior approach and obtains ideal correction as well as reconstruction. We evaluated the new approach systemically to find the advantages and disadvantages on symptom relief, clinical indexes change, kyphosis angle change, the safety and complications of the operation. And we hope that other surgeons can get inspiration from this study to find more effective treatments and thus change the current situation of spine tuberculosis.

## Data and Methods

### *Inclusion and Exclusion Criteria*

#### *Study Design*

This retrospective clinical trial was conducted at the Department of Orthopaedics in the Second Hospital of Shanxi Medical University from July 2012 to December 2017. It was approved by the Institutional Review Board of our hospital. All patients provided written informed consent for the study and surgery. A total of 36 patients were enrolled and conducted primary posterior debridement combined with osteotomy parallel to the endplates. The researchers who enrolled the participants and analyzed the data did not take part in patient care and assessment. Research data were collected on preoperative investigations, blood loss, blood products transfused during surgery, and postoperative investigations.

#### *Inclusion Criteria*

The inclusion criteria for this study are: (i) definite diagnosis of spinal tuberculosis of GATA III; (ii) local Cobb angle of kyphosis greater than or equal to 20°; (iii) the scope of lesions involving three segments or less with or without psoas major abscess; (iv) all the patients receiced primary posterior debridement combined with osteotomy parallel to the endplates for reconstruction; (v) the patients were willing to participate after giving written informed consent; and (vi) complete clinical data of the patient was obtained.

#### *Exclusion Criteria*

The exclusion criteria for this study are: (i) multiple spinal tuberculosis; (ii) the widespread abscess anterior to the vertebral body involving multi‐segments; (iii) active tuberculosis; and (iv) patients undergoing anterior surgery or anterior combined posterior approach surgery.

### *General Information*

**Figure 1:**
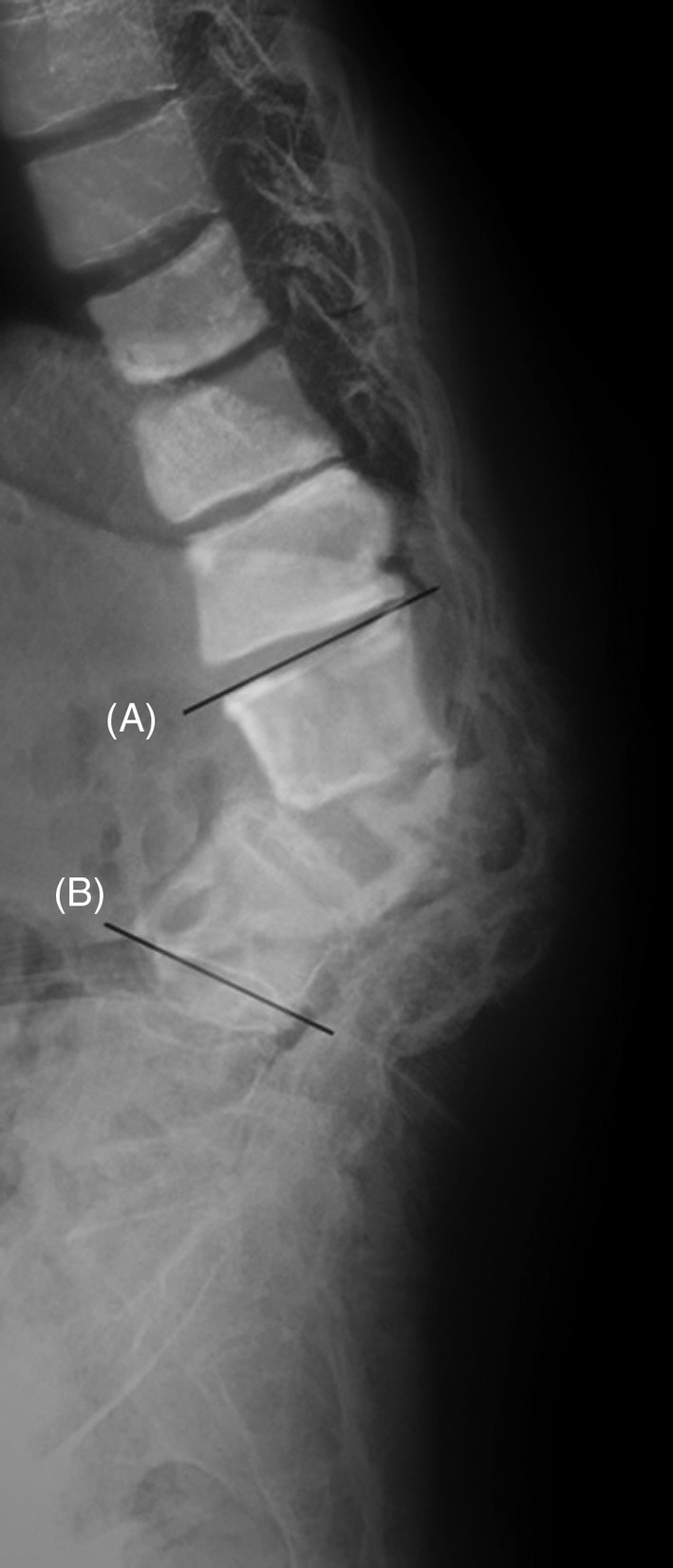
The Angle formed by line (A) and line (B) is posterior convex Cobb angle.

We analyze 53 cases of thoracic and lumbar spinal tuberculosis of GATA III from July 2012 to December 2017 retrospectively, which were all recorded at the Second Hospital of Shanxi Medical University. According to the exclusion criteria, a total of 36 cases have been included in this study. There were 16 males and 20 females (19–72 years of age, averaging 38.8 years old). The duration of disease ranged from 5 to 21 months, with an average of 10.6 months. There were 15 cases of thoracic segments, 12 cases of thoracolumbar segments (T_11_‐L_2_) and nine cases of lumbar segments. The kyphosis angle was 23°–55° (averaged 37.21° ± 3.28°). The kyphosis Cobb angle was formed by the upper endplate of the upper vertebral body and the lower endplate of the next vertebral body on the lateral X‐ray (Fig. 1). Visual analogue scale (VAS) scores of back pain on admission were 0–8 points (averaged 5.58 ± 1.66 points). One patient had no back pain but only a low back mass. Eight patients were associated with neurological dysfunction in both lower extremities. ASIA grade C included three patients, grade D for five patients and grade E for 28 patients. Seven patients (19.4%, 7/36) had typical tuberculosis symptoms such as low fever, night sweats, and fatigue. None of the 36 patients had active pulmonary tuberculosis. At admission, ESR was 21‐110 mm/1 h (55.34 ± 1.72 mm/1 h) and CRP was 18‐68 mg/L (35.22 ± 2.46 mg/L). The imaging findings of 26 patients showed typical single intervertebral space involvement. Eight showed two intervertebral spaces involvement, and two with three intervertebral spaces involvement. All the patients have paravertebral abscess, among which six patients had huge abscesses and fluid flow.

### *Preoperative Preparation*

Standard four‐combining chemotherapy regimen (isoniazid 0.3 g/d, rifampin 0.45 g/d, ethambutol 0.75 g/d, pyrazinamide 0.75 g/d) was used for anti‐tuberculosis treatment for 2–3 weeks preoperatively, and nutritional support therapy was strengthened to correct anemia. General condition of the patients improved with body temperature < 37.5°, hemoglobin > 100 g/L, and erythrocyte sedimentation rate (ESR) < 50 mm/1 h. Surgical treatment was performed when no other surgical contraindication had been observed.

### *Surgical Methods*

Endotracheal intubation combined with general anesthesia was used. The patients were placed in the prone position.

After location with C‐arm X‐ray fluoroscopy, posterior median incision was taken to expose the lesioned segment and its upper and lower 2–3 normal segments. Pedicle screws were instrumented into the normal vertebra first. Only if the upper part of the vertebra was not violated, it could be instrumented. C‐arm fluoroscopy was performed to ensure the pedical screws in ideal position. When both sides (the left and right sides of the lesion) were needed to be debrided, the side with severer destruction was selected for osteotomy and implantment first. After procedure side was determined, the contralateral connecting rod was installed for temporary fixation to keep kyphosis in situ or in a mild orthopaedic state.

The spinous process, lamina and articular processes of the diseased vertebra could be resected. For the thoracic vertebra, the corresponding costal transverse joint and the rib head could be removed for larger exposure. The intercostal nerve could be cut off. Then, enter the focus from the rear. The dural sac could be retracted slightly in the lumbar spine, which made entering point more inclined to the center. The dead bone, caseous necrosis, inflammatory granulation and abscesses were thoroughly removed. If the paravertebral abscess was formed, the aspirator tube could be inserted into the abscess cavity to suck it up. Then, the elbow curette was inserted into the cavity further to scrape the inflammatory granulation tissues on the wall. For giant abscess in psoas major, the entrance leading to the abscess cavity should be found or psoas could be separated bluntly until the abscess cavity was seen. Silicone catheter was inserted into the cavity to suck out pus, and the cavity was repeatedly rinsed with saline until it was cleared. The vertebral lesion was scraped till the healthy bone surface was seen. If the contralateral lesion cannot be cleaned, we can change the fixation to deal with it. After debridement the osteotomy can be performed. There were three most common types of vertebral destruction: (i) when the intervertebral space is mainly affected and adjacent vertebras are partly affected; (ii) when the vertebra body but also upper or lower intervertebral space is affected; and (iii) where the destruction extends infection to multiple segments and becomes extensive. Regardless of whatever type of destruction, just follow one principle: osteotomy is only for the most upper and lower incompletely destroyed vertebra. And it should be ensured that the superior endplate of the upper vertebral body and inferior endplate of the lower body is undamaged. For the upper body, osteotomy was performed parallel to its superior endplate and the resected surface was consistent with the highest point of focus. For the lower body, osteotomy was performed parallel to its inferior endplate and the resected surface was consistent with the lowest point of focus. For the vertebrae with less destruction and more complete endplate, the osteotomy is not necessary. Scraping the cartilage and hardened bone until the surface errhysis is enough. For some skip lesions, do the same procedure to each site respectively.

**Figure 2:**
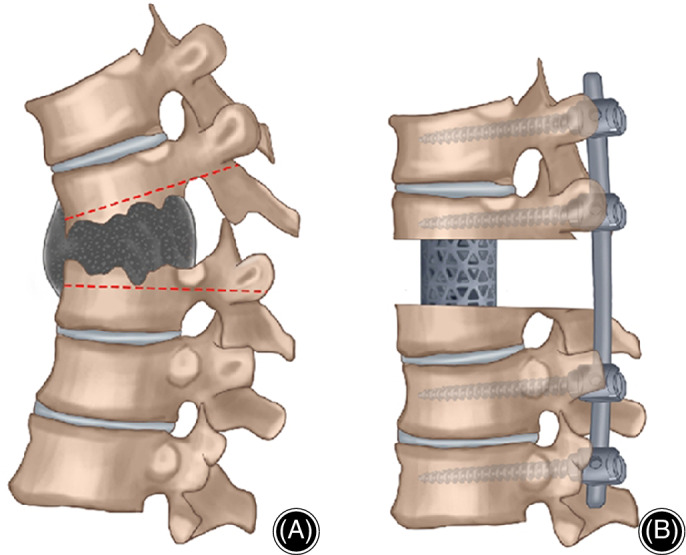
The red line in figure (A) is the osteotomy plane, and (B) shows the titanium mesh implantation after osteotomy and orthopedic surgery while the pedicle screws were fixed.

When the osteotomy was completed, the kyphosis deformity was then corrected by re‐installation of bilateral fixation rods. For severe deformity, alternate installation and progressive orthopaedic repair were required. After correction, the intervertebral height was measured and titanium mesh was trimmed to match it. The titanium mesh filled with autogenous or allogeneic bone was then implanted into the focus (Fig. 2). The position of the titanium mesh is advised to be the center of the vertebra with slightly inclined to the front. After the positive and lateral C‐arm fluoroscopy confirmed the satisfactory position of titanium mesh, it was then fixed with appropriate pressure. Drainage tube was placed, the lesion tissues were kept for pathological examination, and the incision was sutured layer by layer.

### *Postoperative Treatment*

The drainage tube was removed when the drainage volume in the surgical area was <50 mL/d. Four‐combining chemotherapy regimen (isoniazid 0.3 g/d, rifampin 0.45 g/d, ethambutol 0.75 g/d, pyrazinamide 0.75 g/d) was continued for 9–12 months. Patients should stay in bed for 1–2 weeks, get out of bed with thoracolumbar support after 2–3 weeks for moderate activities, do strengthening exercises after 3 months, and the support can be removed after 6 months for normal activities. Reexamination of ESR, CRP, as well as liver and kidney function monthly for 3 consecutive months after surgery, and then, reexamination was conducted every 3 months.

### *Methods and Contents of Follow‐up*

The X‐ray films in the positive and lateral position were taken within one week after surgery. Patients were told to go to the outpatient care for re‐evaluation in the 1^st^, 3^rd^, 6^th^, 9^th^, and 12^th^ month post‐operation, and then once every 6 months until reaching the standard cure. This standard recovery state involves: (i) the symptoms have no recurrence during the 6 months after operation; (ii) ESR is in the normal range; (iii) X‐ray film shows the lesional vertebra reaches osseous healing; and (iv) patients return to normal activities and light work for 3–6 months. The re‐evaluation includes ESR, CRP, ASIA grading of nerve function, test of liver and kidney function, positive and lateral X‐ray films, and additional computed tomography (CT) for some patients.

The definitive explanation of the measurement methods are as follows:

#### *VAS*

VAS is a index used to evaluate the pain level of patients. Patients can use the number 0–10 to define their pain: 0–2 means comfortable; 3–4 means mild discomfort; 5–6 means moderate discomfort; 7–8 means severe discomfort; and 9–10 means extreme discomfort.

#### *ASIA Grading System*

ASIA grading system is used to describe the damage degree of the neurological function. Grade A means the patient has no sensory and motor function below the damage level. Grade B means the patient has sensory function but no motor function below the damage level. Grade C means the patient has motor function, but the force of the key muscles is below level 3. Grade D means the force of the key muscles is above or equal to level 3. Grade E means the patient has normal sensory and motor function. The degree of the force of key muscles is devided into five levels. Level 0 means complete paralysis. Level 1 means the contraction of muscles can be touched. Level 2 means the patient can use the joint actively but cannot use it against gravity. Level 3 means the patient can use the joint actively against gravity. Level 4 means the patient can use the joint actively against moderate resistance. Level 5 means normality.

#### *Cobb Angle*

The angle is formed by the line of the upper endplate and that of the lower endplate of end vertebraes, which is also called the posterior convex angle. The end vertebraes refer to the upper and lower vertebras with maximum inclination of the kyphosis (Fig. 1).

### *Statistical Analysis*

SPSS13.0 statistical software (SPSS, USA) was used for statistical analysis. Measurement data were expressed as mean ± standard deviation. VAS score, Cobb angle of the lesional segment, ESR, and CRP values pre‐ and post‐operation were compared with that of the last follow‐up by one‐way ANOVA and repeated measures ANOVA. *P*‐value <0.05 was considered significant difference for all tests.

## Result

### *Operation Condition*

The average operation time was 3.8 h (3.0–5.5 h), blood loss was 730 mL (500–1200 mL), and blood transfusion was 620 mL (400–1000 mL). There was no injury of great vessels occurring during the operation, no cerebrospinal fluid leakage caused by dural sac tear, and also no death.

### *Clinical Efficacy*

**Figure 3:**
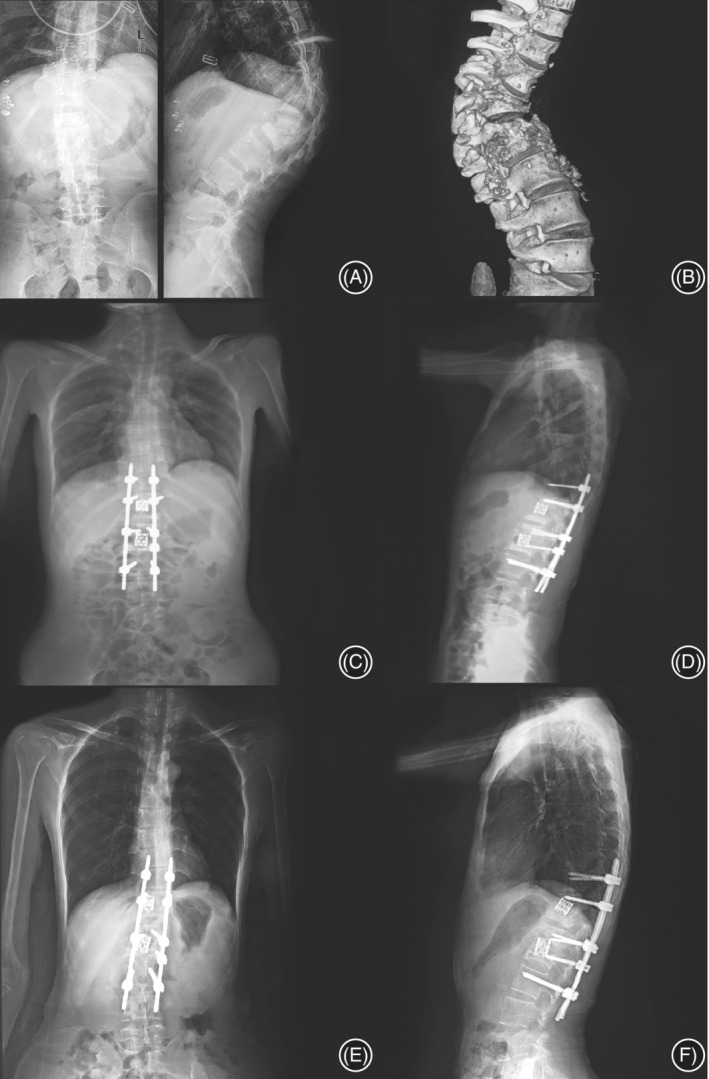
Female, 31 years old, spine tuberculosis of T_11__‐12_ and L_1‐2_ with bilateral paravertebral abscesses: (A). Preoperative positive and lateral radiographs showed the destruction of the intervertebral space of T_11‐12_ and L_1‐2_ with the formation of kyphosis. (B) Three‐dimentional reconstruction of computerized tomography (CT) clearly showed severe bone destruction in the anterior and middle column. (C, D) Postoperative positive and lateral X‐ray films showed that the correction of spinal deformity was ideal, the titanium mesh was in a moderate position, and the fixation was firm. (E, F) 1 year after surgery, the positive and lateral X‐ray films showed that the spinal orthopedics maintained well, the titanium mesh was free from loosening and sinking, and the internal fixation was firm.

Patients were followed up for 12 to 24 months, with an average of 16 months. During follow‐up, no loosening, shedding or fracture of internal fixation was found in all patients (Fig. 3). By the last follow‐up, all patients' symptoms such as low fever, night sweats, weight loss, and fatigue recovered completely. All the 36 patients returned to normal activities 12 months after the operation.

#### *VAS*

VAS score improved from 5.58 ± 1.66 points (0–7 points) before surgery to 3.25 ± 0.92 one month after surgery and 2.12 ± 0.73 at the last postoperative follow‐up. It was shown that there was statistical difference (*P* = 0.01) pre‐ and post‐operation, which illustrated significant pain relief.

#### *ASIA Grading System*

Two patients of ASIA grade C recovered to grade E and one recovered to grade D. All the five patients of grade D recovered to grade E (Table 1). These indicators showed that almost all of the patients who underwent the surgery in our study achieved the desired functional recovery, which indirectly proved the thoroughness and efficiency of the operation.

**Table 1:** Neurological function of patients before and after surgery

Preoperative ASIA classification	The number of cases	At the last follow‐up, ASIA was graded				
		A	B	C	D	E
A	0	0	0	0	0	0
B	0	0	0	0	0	0
C	3	0	0	0	1	2
D	5	0	0	0	0	5
E	28	0	0	0	0	28

#### *Cobb Angle*

The preoperative kyphosis angle improved from 37.21°± 3.28° (22°–55°) to 5.72° ± 2.66° one month after surgery and 5.99°± 1.92° at the last postoperative follow‐up, and the average correction rate of kyphosis deformity was 83.4% (Table 2). It was shown that there was statistical difference (*P* = 0.01) pre‐ and post‐operation, which proved that the operation has good orthopaedic function.

**Table 2:** Improvement of efficacy indicators before and after treatment (mean±SD)

Times	VAS (points)	Cobb angle (°)	ESR (mm/1 h)	CRP (mg/L)
Preoperative	5.58 ± 1.66	37.21 ± 3.28	55.34 ± 1.72	35.22 ± 2.46
One month after surgery	3.25 ± 0.92	5.72 ± 2.66	28.22 ± 3.76	12.67 ± 2.82
Last follow‐up	2.12 ± 0.73	5.99 ± 1.92	11.54 ± 0.46	4.50 ± 2.11
*F*‐value	175.15	55.23	187.61	114.34
*P* value	0.01	0.01	0.02	0.02

#### *ESR and CRP*

The ESR recovered from 55.34 ± 1.72 mm/1 h before surgery to 28.22 ± 3.76 mm/1 h 1 month after surgery and 11.54 ± 0.46 mm/1 h at the last follow‐up. CRP recovered from 35.22 ± 2.46 mg/L before operation to 12.67 ± 2.82 mg/L 1 month after surgery and 4.50 ± 2.11 mg/L at the last follow‐up. It was shown that there was respective statistical difference (*P* = 0.02 for ESR and *P* = 0.02 for CRP) pre‐ and post‐operation, which proved that the operation is effective in removing the lesion and controlling the infection.

### *Postoperative Complications*

One case had subcutaneous tuberculous effusion one month after surgery. The second surgical exploration confirmed that the effusion was only confined to the subcutaneous area and was not connected with the original tuberculosis focal area. There was no recurrence after re‐debridement. One patient who suffered spinal cord shock caused by osteotome vibration showed transient paraplegia, but his/her neurological function began to recover 12 h after the operation, and recovered completely 3 months later.

## Discussion

### *Advantages and Disadvantages of Simple Posterior Approach Surgery for Spinal Tuberculosis*

Most spinal tuberculosis destroy the anterior column structure of the spine, while the posterior appendices are rarely affected. Therefore, for a long time, the anterior approach surgery was ‘golden standard’ for treatment of spinal tuberculosis^8^. However, the anterior approach has many complications such as large trauma and high surgical risk. In cases of multiple lesions with bilateral abscesses, the anterior approach is often difficult to achieve the purpose of debridement. In addition, the anterior approach makes poor deformity correction for kyphosis. With the improvement of internal fixation instruments and surgical tools, as well as the improvement of posterior surgical techniques, one‐stage posterior approach is increasingly applied to the treatment of spinal tuberculosis. Compared with the anterior approach, the one‐stage posterior approach has relatively less trauma and makes it easy to enter the lesion due to fewer anatomical layers and no important organs nearby. Therefore, this approach causes fewer complications. In addition, pedicle fixation in the posterior approach can provide better biomechanical stability than anterior internal fixation. Thus, the posterior approach makes the surgical correction effect more ideal and the fixation more stable^9^, ^10^.

Because of the large destruction scope and severe kyphosis deformity, the single posterior surgery has obvious advantages for spine tuberculosis of GATA III. Not only can it remove a wide range of abscess, dead bone, and caseous necrosis, but it can also correct the kyphosis deformity maximumly. Due to the block caused by the anterior sternum and mediastinal tissues, the anterior approach is difficult for cervicothoracic lesions for narrow vision and higher surgical risk. Furthermore, the smaller vertebral bodies and poor quality after erosion make the screw set and correction for cervicothoracic vertibra both difficult^11^. Therefore, the posterior approach is more advantageous for cervicothoracic tuberculosis. With the development of technology, the psoas major abscess, gravitation abscess, and jumping lesions, which were previously considered as contraindication of the posterior approach, can now be well cleared by the posterior approach alone.

Because spinal tuberculosis violates the anterior column more commonly, the front tends to leave a larger defect after debridement and deformity correction, which usually requires titanium mesh for reconstruction. However, the destruction of lesions is always irregular and the residual vertebra is ossified which made it difficult to implant mesh and keep it stable in the traditional posterior approach. The main problems are as follows: firstly, the titanium mesh implantation cannot guarantee the effective height; secondly, the titanium mesh can not always provide effective support between the irregular vertebrae, which will affect the stability of reconstruction; thirdly, the two ends of the titanium mesh cannot make full contact with the vertebral bone surface, which is bound to affect the fusion between the vertebral bodies.

### *Technical Features and Advantages of Osteotomy Parallel to the Endplates*

Osteotomy parallel to the endplates makes the surfaces of the implantation area flat by resecting the remaining irregular parts of the vertebral body, which is not only conducive to the implantation of titanium mesh but also convenient to adjust the mesh position to ensure its stability. What's more,the exposure of the fresh bone surfaces caused by resection of the harden bone will further accelerate the vertebral fusion. The osteotomy can also make the implant channel unobstructed. As a result, measure for mesh height becomes easier, then the ideal mesh with appropriate thickness andheight can be chosen to increase the stability and contact area, and getting the right height of the segments can avoid excessive tensile or shortening of the spinal cord, reducing complications of the corresponding nerve injury^12^.

Osteotomy parallel to endplates does not pursue the complete removal of the entire transverse surface of the vertebral body. The main purpose of it is to ensure the effective implantation, support and fusion. Therefore, the range of the osteotomy is just sufficient for ideal placement of mesh.

### *Potential Risks of Osteotomy Parallel to the Endplate*

Osteotomy parallel to the end plate can maximally resect the sclerotic bone formed by tuberculosis lesions, which is conducive to fusion. However, due to the difficulty in the osteotomy of the sclerotic bone, excessive force always needs to be applied. This can make relatively severe vibration that is dangerous for the spinal cord, especially in thoracic segments, that can cause neurological dysfunctions. For this reason, more attention should paid to the thoracic lesions. In this group, there was one case in the T_10‐11_ segment which recorded transient paralysis due to the vibration, and it took 3 months to fully recover. Therefore, it is recommended to select sharp osteotome and conduct the procedure gently and patiently. If necessary, ultrasonic osteotome or abrasive drilling is recommended. The risk of spinal cord shock or contusion can be reduced by opening the spinal canal as completely as possible and leaving the spinal cord free before osteotomy. Osteotomy is usually carried out in the vertebral cancellous parts and the titanium mesh is inevitably placed between the cancellous bone rather than the end plates. But in the cases of this study, the subsidence of the mesh occurred rarely, which may be related to the bone sclerosis caused by tuberculosis. Although a part of the sclerotic bone is resected, the remaining part can still provide an effective support.
